# Cancer therapy resistance from a spatial‐omics perspective

**DOI:** 10.1002/ctm2.70396

**Published:** 2025-07-17

**Authors:** Yinghao Zhang, Cheng Yang, Xi Chen, Liang Wu, Zhiyuan Yuan, Fan Zhang, Bin‐Zhi Qian

**Affiliations:** ^1^ Department of Oncology Shanghai Medical College, The Human Phenome Institute, Zhangjiang‐Fudan International Innovation Center, Center for Integrative Spatial‐Omics Research, Fudan University, Fudan University Shanghai Cancer Center Shanghai China; ^2^ Shanghai Innovation Institute Shanghai China; ^3^ Shanghai Academy of Artificial Intelligence for Science Shanghai China; ^4^ Department of Orthopedic Oncology Changzheng Hospital Second Military Medical University Shanghai China; ^5^ State Key Laboratory of Genome and Multi‐omics Technologies, BGI Research Shenzhen China; ^6^ Institute of Science and Technology for Brain‐Inspired Intelligence MOE Key Laboratory of Computational Neuroscience and Brain‐Inspired Intelligence MOE Frontiers Center for Brain Science Fudan University Shanghai China; ^7^ Center for Medical Research and Innovation Shanghai Pudong Hospital, Fudan University Pudong Medical Center, Fudan University Shanghai China; ^8^ Department of Chemistry State Key Laboratory of Molecular Engineering of Polymers and iChem Shanghai Key Laboratory of Molecular Catalysis and Innovative Materials Fudan University Shanghai China

**Keywords:** cancer therapy resistance, spatial omics

## Abstract

Cancer therapy resistance (CTR) remains a significant challenge in oncology. Traditional methods like imaging, liquid biopsies and conventional omics analyses provide valuable insights, but lack the spatial resolution to fully characterise heterogeneity of tumour and the tumour microenvironment (TME). Recent advancements in spatial omics technologies offer unprecedented insights into the spatial organisation of tumours and TME. In this review, we summarise current methodologies for CTR research and highlight how spatial omics technologies and computational methods are revolutionising our understanding of CTR mechanisms. We also summarise recent studies leveraging spatial omics to uncover novel insights into CTR across various cancer types and therapies and discuss future opportunities.

## Graphical abstract

Spatial omics technologies reveal unprecedented insights into cancer therapy resistance (CTR) by resolving spatial heterogeneity of tumour and microenvironment. This review highlights cutting‐edge spatial omics and computational technologies, showcasing their transformative potential in deciphering CTR mechanisms and uncovering novel diagnostic and therapeutic opportunities.

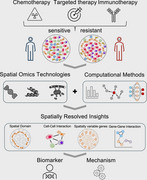

## 1 INTRODUCTION

Cancer remains a major clinical challenge worldwide with over 10 million deaths annually^1^ and is projected to incur a global economic cost of $25.2 trillion (in 2017 international dollars) from 2020 to 2050, according to a macroeconomic analysis of 29 cancer types across 204 countries.^2^ Although chemotherapy, targeted therapy and immunotherapy have revolutionised cancer treatment over the decades, their success is limited by therapy resistance.^3^, ^4^, ^5^, ^6^, ^7^, ^8^ Cancer therapy resistance (CTR) refers to the stage where cancer develops the ability to withstand treatment, resulting in treatment failure—a critical issue that reduces quality of life and increases mortality.^9^ CTR can be intrinsic, present before treatment, or acquired, developed during or after treatment. It can also manifest as primary resistance, where the cancer is unresponsive to initial therapy, or secondary resistance, where cancer initially responds but later progresses despite continued treatment.^10^ Many molecular mechanisms have been identified to contribute to CTR, which has been reviewed extensively.^9^

Current CTR research relies on three core methodologies: (1) medical imaging, including computed tomography, magnetic resonance imaging and positron emission tomography, to monitor tumour growth; (2) liquid biopsy to detect circulating biomarkers such as circulating tumour DNA and tumour‐derived extracellular vesicles and (3) bulk or single‐cell omics of tumour specimens decoding molecular biomarkers and resistance mechanisms. Each offers unique advantages but also critical limitations. While imaging non‐invasively tracks structural changes at organ scale, it lacks molecular granularity; liquid biopsy enables dynamic monitoring of resistance biomarkers but suffers from tissue context loss; conventional omics reveals molecular networks yet fails to preserve the spatial architecture of the tumour and its microenvironment. This spatial gap persists despite advances in digital pathology and conventional omics, underscoring the need for technologies that concurrently map molecular landscapes and cellular ecosystems within native tumour microenvironment (TME).

Recent advances in spatial omics technologies begin to illustrate many novel CTR mechanisms, particularly in terms of tumour and microenvironment heterogeneity. Technological advances in spatial transcriptomics, proteomics and metabolomics have been comprehensively reviewed recently.^11^, ^12^, ^13^, ^14^, ^15^, ^16^, ^17^ In the current review, we introduce the current methodology for CTR research, advancement of spatial omics technologies and computational methods, summarise recent spatial omics studies focusing on CTR and discuss future directions or opportunities.

## 2 SPATIAL OMICS TECHNOLOGIES

Understanding the mechanisms of CTR requires not only identifying involved molecules, but also determining the spatial context of these molecules in TME. Spatial omics technologies fulfil this need by preserving tissue architecture while capturing molecular information at high resolution. Spatial omics technologies address this need by preserving tissue architecture while enabling high‐resolution molecular profiling. To facilitate the application of spatial omics in CTR research, we provide a summary of the most commonly used commercial platforms for spatial transcriptomics and proteomics, along with their key features (Table 1).

**TABLE 1:** Commercialised spatial omics technology.

Omics type	Strategy	Technology	Sample type	Spatial resolution	Detection area	RNA plex	Protein plex
Spatial transcriptomics	Sequencing based	10X Genomics Visium	FF, FFPE	55 µm	6.5 mm × 6.5 mm (11 × 11 mm)	Whole transcriptomics	–
		NanoString GeoMx DSP	FF, FFPE, TMA, fixed cell culture	10–600 µm	14.6 mm × 36.2 mm	Whole transcriptomics	580
		BGI Genomics Stereo‐seq	FF, PFA fixed	0.5 µm	13 cm × 13 cm	Whole transcriptomics	–
		Curio Biosciences Seeker	FF	10 µm	Whole slide	Whole transcriptomics	–
		Curio Biosciences Trekker	FF	Single cell	Whole slide	Whole transcriptomics (single‐nucleus)	–
		10X Genomics Visium CytAssist	FF, FFPE	55 µm	6.5 mm × 6.5 mm (11 mm × 11 mm)	Whole transcriptomics	35
		10X Genomics Visium‐HD	FF, FFPE	2 µm	6.5 × 6.5 mm	Whole transcriptomics	–
	Imaging based	Vizgen MERSCOPE	FF, FFPE	0.1 µm	1 cm × 1 cm	1000	–
		Vizgen MERSCOPE ULTRA	FF, FFPE, adherent or suspended cells	≤0.02 µm	1.25 cm^2^/3 cm^2^	1000	–
		NanoString CosMx SMI	FF, FFPE, TMA	0.05–0.12 µm	3 cm^2^	6000+	68+
		10X Genomics Xenium	FF, FFPE	0.05 µm	12 mm × 24 mm (recommend: 10.45 mm × 22.45 mm)	400+ (theoretical up to 5000)	–
Spatial proteomics	Cyclic microscopy	NeoGenomics MultiOmyx	FF, FFPE	Subcellular	Whole slide (4 µm thickness)	–	60
		Lunaphore COMET	FF, FFPE	0.28 µm	12.5 mm × 12.5 mm	12 RNA+ 24 protein or 4 RNA +28 protein	40
		Miltenyi MACSima	FF, FFPE	Subcellular	Whole slide	27	200+
		Akoya Bioscience PhenoCycler‐Fusion (formerly CODEX)	FF, FFPE	0.25–1 µm	1.6 cm × 1.6 cm	–	100+
	Mass spectrometry based	Standard BioTools Hyperion imaging system	FF, FFPE	1 µm	15 mm × 55 mm	–	46+
		IONpath MIBIscope	FF, FFPE	0.250 µm	2 cm^2^	–	40+

Abbreviations: FF, fresh‐frozen; FFPE, formalin‐fixed paraffin‐embedded; PFA, paraformaldehyde; TMA, tissue microarray.

*Spatial transcriptomics* employs two primary approaches: sequencing based and imaging based. Sequencing‐based spatial transcriptomics utilises barcoded arrays that capture RNA molecules directly from tissue sections, mapping gene expression to specific spatial coordinates through subsequent sequencing. Key commercial technologies include Visium, Visium CytAssist and Visium‐HD (10X Genomics, Pleasanton, CA, USA),^18^ GeoMx Digital Spatial Profiler (GeoMx DSP; NanoString Technologies, Seattle, WA, USA),^19^ Stereo‐seq (BGI Genomics, Shenzhen, China)^20^ and Seeker and Trekker (Curio Biosciences, Palo Alto, CA, USA).^21^, ^22^ Imaging‐based methods tag RNA transcripts directly within tissues using fluorescent probes, visualising their precise location through high‐resolution microscopy. Notable technologies in this category include MERSCOPE and MERSCOPE ULTRA (Vizgen, Cambridge, MA, USA),^23^ CosMx Spatial Molecular Imager (CosMx SMI; NanoString Technologies, Seattle, WA, USA)^24^ and Xenium (10X Genomics, Pleasanton, CA, USA).^25^

Notably, recent advancements in sequencing‐based spatial transcriptomics have substantially improved spatial resolution, evolving from traditional multi‐cell resolution approaches (e.g., Visium, GeoMx DSP) to platforms capable of achieving single‐/sub‐cellular resolution (e.g., Visium‐HD, Stereo‐seq, Curio Seeker/Trekker). These advances have significantly narrowed or even eliminated the resolution gap between sequencing‐based and imaging‐based methods, enabling increasingly precise spatial mapping of gene expression.

*Spatial proteomics* predominantly relies on antibody‐based detection, differentiated primarily by microscopy‐based and mass spectrometry‐based strategies. Microscopy‐based spatial proteomics involves cyclic antibody labelling, imaging and signal removal or inactivation, enabling high multiplexing and subcellular resolution. Prominent commercial platforms include MultiOmyx (NeoGenomics Laboratories, Fort Myers, FL, USA),^26^ COMET (Lunaphore Technologies, Tolochenaz, Switzerland),^27^ MACSima (Miltenyi Biotec, Bergisch Gladbach, Germany)^28^ and PhenoCycler‐Fusion (formerly CODEX, Akoya Biosciences, Marlborough, MA, USA).^29^, ^30^ Mass spectrometry‐based proteomics uses metal isotope‐tagged antibodies analysed through mass spectrometry, offering multiplexed detection with robust spatial resolution. Key platforms include the Hyperion Imaging System (Standard BioTools, South San Francisco, CA, USA)^31^ and MIBIscope (IONpath, Menlo Park, CA, USA).^32^

*Spatial metabolomics* technologies mainly involve mass spectrometry imaging (MSI), which includes two primary approaches: matrix‐assisted laser desorption ionisation (MALDI) and desorption electrospray ionisation (DESI). MALDI‐based approaches involve applying a matrix onto tissue sections, where it co‐crystallises with metabolites. Laser irradiation causes ionisation of the matrix and subsequent desorption and ionisation of metabolites. This technology is particularly suitable for visualising lipids and can achieve high spatial resolution with a maximum resolution of 5 µm.^33^ Commercial technologies in this category are built around high‐resolution mass analysers, such as MALDI–TOF/TOF and MALDI–Orbitrap. DESI‐based approaches utilise a charged solvent spray to ionise metabolites directly from tissue surfaces without the need for a matrix, making it particularly advantageous for small molecule metabolites. DESI–MSI enables faster sample preparation and allows direct tissue analysis under ambient conditions. While its spatial resolution is generally lower than that of MALDI. Recent advancements in nano‐DESI have brought its resolution closer to that of MALDI. Commercial DESI technologies often involve high‐resolution mass spectrometers such as DESI–Q‐TOF and DESI–Orbitrap systems. The choice between MALDI–MSI and DESI–MSI depends on specific biological questions, type of metabolites and desired spatial resolution, with both approaches offering complementary advantages for the spatial mapping of metabolomic distributions within tissues. For more technical details about spatial metabolomics technologies, please refer to a recent review by Planque et al.^16^

Given the breadth and technical heterogeneity of spatial omics technologies, selecting an appropriate platform for studying CTR requires careful alignment between experimental design and the underlying biological context. Key parameters such as spatial resolution, tissue preservation method (for example, fresh frozen versus FFPE), molecular modality (such as transcriptome or proteome) and the resistance mechanism of interest (including clonal selection, immune evasion or stromal remodelling) should be evaluated in relation to the specific research objective. Sequencing‐based spatial transcriptomics platforms offer unbiased, whole‐transcriptome coverage and are well suited for surveying global transcriptional shifts across the TME, particularly when investigating broad changes in resistant subclones or stromal adaptation. However, these methods typically capture RNA from multicellular regions, and their effective resolution is constrained by spot diameter and RNA diffusion, which can limit the ability to resolve fine‐scale cellular interactions.^34^ In contrast, imaging‐based platforms such as CosMx SMI, Xenium and MERSCOPE achieve true single‐cell and subcellular resolution through in situ transcript detection, making them ideally suited for mapping spatially localised resistance programs, particularly those mediated by immune cell exclusion or confined to distinct microenvironmental niches. Tissue preservation further shapes platform choice: FFPE‐compatible platforms are indispensable for retrospective clinical studies, while fresh‐frozen specimens enable transcriptome‐wide discovery with high‐fidelity RNA capture. For resistance mechanisms driven by protein‐level regulators, spatial proteomics or multimodal platforms that co‐detect RNA and protein within the same tissue context can offer critical additional insight. Ultimately, platform selection should reflect a principled match between the spatial and molecular granularity required and the biological scale of the resistance mechanism under investigation, whether the goal is to map rare resistant clones, to profile spatial gradients associated with TME remodelling or to resolve cell–cell interactions that mediate therapeutic escape.

## 3 COMPUTATIONAL METHODS FOR SPATIAL OMICS ANALYSIS

**FIGURE 1:**
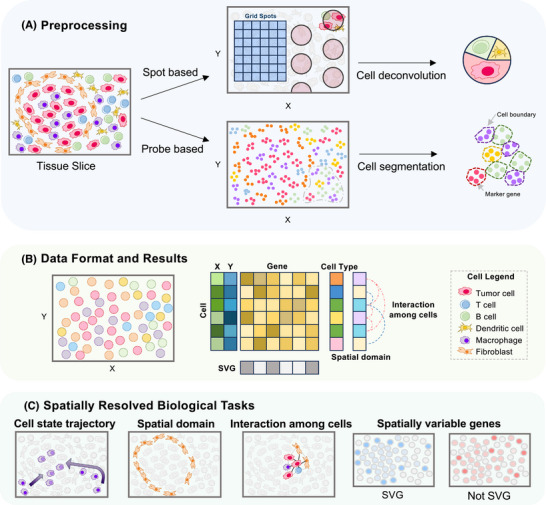
Spatial transcriptomics data analysis workflow for cancer therapy resistance schematic outline of key computational steps in of spatial omics data analysis for CTR research. (A) Preprocessing: Multi‐cell resolution technologies (e.g., Visium, GeoMx DSP) typically require cell type deconvolution, whereas single‐cell or sub‐cellular resolution technologies (e.g., MERSCOPE, Xenium, Stereo‐seq) employ cell segmentation to delineate individual cells. (B) Data format and results: The spatial coordinates (X, Y) of cells are integrated with gene expression data to determine cell identity and spatial domain annotations. Interactions among cells are inferred to reveal tumour‐microenvironment crosstalk. (C) Spatial resolved biological tasks: Multiple downstream analyses, including cell state trajectory analysis, identification of spatial domains, interaction among cells and detection of spatially variable genes (SVGs), can be used to provide insights into potential spatial heterogeneity and crosstalk associated with CTR.

Understanding CTR through spatial omics data requires sophisticated computational methods, including data preprocessing and data mining, capable of handling large datasets with spatial information. Several reviews have summarised these computational methods and provided practical guidelines.^11^, ^35^, ^36^ Considering the broad applications of spatial transcriptomics and the rapid advancement of associated computational tools, many of which are also applicable to spatial proteomics, here we primarily focus on computational methods developed for spatial transcriptomics data analysis, outlining a representative analysis workflow and highlighting key tools and their applications (Figure 1, Table 2).

**TABLE 2:** Computational methods for spatial omics data analysis.

Task	Tools	Application
Cell segmentation	CelloType,^37^ ClusterMap,^38^ SSAM,^39^ Baysor,^40^ GeneSegNet,^41^ RedeFISH,^42^ SCS^43^ and BIDCell^44^	Segmentation of cells for image based spatial data
Cell type annotation	Spatial‐ID,^47^ Tangram,^48^ TACCO^49^ and DestVI^50^	Annotation at single‐cell or sub‐cellular resolution
Cell type deconvolution	CARD,^51^ cell2location^52^ and Tangram^48^	Resolves cell‐type composition at multi‐cell resolution
Multi‐slice integration and alignment	BASS,^55^ SpaDo,^56^ and MENDER^57^ & SANTO,^59^ STalign,^60^ GPSA,^61^ PASTE^62^ and Spateo^58^	Harmonising gene expression across slices, reducing batch effects, building unified views and mapping slices into common coordinate systems
Spatially variable genes	Gitto k‐means,^65^ Gitto rank,^65^ MERINGUE,^66^ nnSVG,^67^ SOMDE, SPARK‐X^69^ and SpatialDE^70^	Reveals spatial differences in gene expression across tissue regions, aiding the identification of localised biological processes
Gene patterns	SpatialDE,^70^ GLISS,^72^ MERINGUE^66^ and stLearn^73^	Maps gene expression patterns within tissues, uncovering regional functions and structures
Spatial domains	SpaGCN,^74^ BayesSpace,^75^ stLearn,^73^ SEDR,^76^ CCST,^77^ SCAN‐IT,^78^ STAGATE,^79^ SpaceFlow,^80^ conST,^81^ BASS,^82^ GraphST,^83^ NicheCompass,^85^ BANKSY,^86^ Taichi^87^ and CytoCommunity^88^	Clusters cells based on spatial and molecular features, identifying tissue microenvironments and cellular communities
Interaction among cells	COMMOT,^89^ SVCA,^90^ SpaOTsc,^91^ GCNG,^92^ Giotto,^65^ MISTy,^93^ DIALOGUE^94^ and stLearn^73^	Uncovers spatial cell–cell interaction networks, elucidating their regulatory roles in tissue function
Interaction among genes	SpaOTsc,^91^ scHOT,^95^ MESSI,^96^ GCNG^92^ and MISTy^93^	Analyses spatial gene–gene interactions to reveal local regulatory networks within specific tissue regions
Cell state trajectories	Pseudo‐time‐space (PSTS) in stLearn,^73^ spaTrack^114^ and SPATA2^97^	Reconstructs spatial trajectories of cell states, providing insights into cell differentiation and evolution in their native environment
End‐to‐end pipelines	PRISM,^98^ SPACEc,^103^ SPEX,^106^ SpatialOne^107^ and ENACT^109^	Integrated platforms combining preprocessing, analysis and visualisation

### 3.1 Data preprocessing

Effective data preprocessing is crucial to ensure that spatial omics data are accurately transformed into usable formats for downstream analysis. In the preprocessing stage, the objective is to transform raw spatial omics data into a cell‐by‐gene matrix with spatial information.

Most commercial spatial omics technologies come with dedicated preprocessing platforms, including Space Ranger for 10X Genomics Visium (HD), DSP GeoMx Data Analysis Suite (DSPDA) Nanostring GeoMx, AtoMx™ Spatial Informatics Platform (SIP) for Nanostring CosMx SMI and Stereo‐seq Analysis Workflow (SAW) for BGI Stereo‐seq among others. However, these tools sometimes fail to meet researchers’ needs, particularly for technologies with single‐cell resolution. In such cases, customised algorithms, particularly on *cell segmentation*, are needed. A thorough comparison is recommended to choose the most suitable method among many options, including CelloType,^1^ ClusterMap,^38^ SSAM,^39^ Baysor,^40^ GeneSegNet,^41^ RedeFISH,^42^ SCS^43^ and BIDCell.^44^

Following cell segmentation, subsequent steps include normalisation, cell type annotation, and, if necessary, multi‐slice integration and alignment.

*Normalisation* of spatial omics data poses unique challenges due to spatial dependencies in gene expression and influences from the tissue microenvironment, which are not adequately addressed by traditional single‐cell RNA sequencing (scRNA‐seq) normalisation methods. Bhuva et al.^45^ demonstrated that library size normalisation often obscures spatial structure and negatively impacts domain identification. Atta et al.^46^ further showed that gene count‐based normalisation in imaging‐based spatial transcriptomics can introduce region‐specific biases, particularly when using skewed gene panels, potentially leading to false positives or negatives. Both studies emphasise the need for specialised normalisation methods that preserve the spatial context of the data. Nonetheless, the field largely continues to rely on these scRNA‐seq methods, underscoring the need for dedicated normalisation strategies.

*Cell type annotation* strategies for spatial omics data differ according to the resolution provided by different technologies. For spatial transcriptomics technologies providing single‐/sub‐cellular resolution (e.g., Visium‐HD, Stereo‐seq, MERSCOPE, CosMx SMI, Xenium), cell type annotation approaches resemble those used in scRNA‐seq, primarily relying on clustering followed by annotation based on marker gene or protein expression profiles. Several automated annotation tools have been developed and widely applied in these scenarios, including Spatial‐ID,^47^ Tangram,^48^ TACCO,^49^ DestVI.^50^ In contrast, for platforms at multi‐cell resolution (e.g., standard Visium, GeoMx DSP), the preferred approach is *cell type deconvolution*, which estimates cellular composition within each spatial location. Commonly employed cell type deconvolution methods include CARD,^51^ cell2location^52^ and Tangram.^48^ Method selection can be guided by findings from recent benchmarking study.^53^

*Multi‐slice integration and alignment* are crucial for building a unified spatial and molecular view of tissues.^54^ *Integration* unifies gene expression across slices, harmonising cell types and spatial domains to reduce batch effects. Methods like BASS,^55^ SpaDo^56^ and MENDER^57^ integrate multi‐slice data through distinct approaches: BASS applies a Bayesian model for joint cell type and domain analysis across slices; SpaDo clusters adjacent cell types spatially; and MENDER uses multi‐range cellular context embeddings to enable integration across large datasets. *Alignment*, on the other hand, maps slices into a common coordinate system (CCS) to address spatial inconsistencies, allowing for accurate 3D tissue reconstruction. Methods like Spateo,^58^ SANTO,^59^ STalign,^60^ GPSA^61^ and PASTE^62^ enhance spatial alignment, enabling more detailed spatial and molecular insights into tissue architecture. While benchmark studies offer useful guidance for method selection,^63^ the rapid emergence of new techniques also invites researchers to explore alternatives such as Spateo.

### 3.2 Down‐stream analysis

In addition to cellular composition, down‐stream analysis of spatial omics data focuses on spatial patterns and spatial interactions. Spatial pattern recognition can be categorised into two types: gene spatial pattern recognition and tissue spatial pattern recognition.^64^ These tasks include identification of spatially variable genes (SVGs), gene patterns, spatial interactions among cells and genes and spatial domains.^11^

*SVGs* elucidate transcriptomic differences within different tissues compartments, which were largely overlooked by previous technologies. Various SVG detection methods have been developed, including Gitto k‐means,^65^ Gitto rank,^65^ MERINGUE,^66^ nnSVG,^67^ SOMDE,^68^ SPARK‐X^69^ and SpatialDE.^70^ A benchmarking study has evaluated these methods based on several criteria: consistency in SVG selection across different methods, reliability of reported statistical significance, accuracy and robustness of SVG detection, performance of selected SVGs in downstream applications, such as clustering of spatial domains, and computational efficiency in terms of time and memory usage.^71^

*Gene patterns* refer to specific spatial distributions of gene expression within a tissue. Analysing gene patterns helps to uncover underlying biological processes, such as developmental pathways, tissue organisation and disease mechanisms. Different SVGs might exhibit distinct spatial patterns, such as gradients, clusters or periodic expressions. Methods such as SpatialDE,^70^ GLISS,^72^ MERINGUE^66^ and stLearn^73^ have been developed to analyse these gene patterns.

*Spatial domains* can be identified through spatial clustering, which is crucial for visualising tissue anatomy, inferring spatial continuity within tissues, detecting domain‐specific marker genes, identifying spatial signatures and domain‐dependent molecular regulatory networks associated with CTR. Recently, Yuan et al. benchmarked 13 computational spatial clustering methods, including SpaGCN,^74^ BayesSpace,^75^ stLearn,^73^ SEDR,^76^ CCST,^77^ SCAN‐IT,^78^ STAGATE,^79^ SpaceFlow,^80^ conST,^81^ BASS^82^ and GraphST,^83^ across 34 spatial transcriptomics datasets (comprising seven distinct datasets).^84^ This benchmarking study concluded that existing methods complement each other in terms of performance and functionality, providing guidance for selecting the most suitable methods for specific scenarios. Researchers may also consider emerging algorithms such as NicheCompass,^85^ BANKSY,^86^ Taichi^87^ and CytoCommunity.^88^ These approaches are specifically designed to delineate fine‐grained cellular ecosystems or microenvironments (niches), enabling a deeper characterisation of tissue organisation and intercellular interactions. Such insights offer significant potential for identifying niche‐specific mechanisms that drive CTR.

*Spatial interaction* among cells and genes within TME is vital for deciphering CTR mechanisms. *Cell–cell interactions* can be inferred by incorporating proximity information among cells. Methods such as COMMOT,^89^ SVCA,^90^ SpaOTsc,^91^ GCNG,^92^ Giotto,^65^ MISTy,^93^ DIALOGUE^94^ and stLearn^73^ have been developed to analyse these interactions, leveraging spatial data to enhance our understanding of cellular dynamics. *Gene–gene interactions* analysis provides insights into tissue‐specific regulatory networks. Methods such as SpaOTsc,^91^ scHOT,^95^ MESSI,^96^ GCNG^92^ and MISTy^93^ facilitate prediction of potential ligand–receptor pairs and identify spatially associated genes. These approaches can uncover potential pathways driving cellular responses and inform therapeutic strategies.

*Spatial cell state trajectories* further extend beyond static spatial features to embrace the dynamic transitions and interactions that drive cellular processes within tissues. By mapping trajectories, researchers can decipher the dynamic transitions and interactions of cellular states within the TME. Methods like pseudo‐time‐space in stLearn,^73^ spaTrack^1^ and SPATA2^97^ reveal these dynamic patterns.

In summary, appropriate computational tools based on specific technology and data type are essential to investigation of CTR mechanisms and identification of diagnostic markers.

### 3.3 End‐to‐end analysis tools

The previous section outlined individual tools for specific spatial omics tasks, such as segmentation and normalisation, requiring manual integration into custom workflows. This approach offers flexibility but increases complexity and demands expertise. End‐to‐end analysis tools, by contrast, provide integrated pipelines combining preprocessing, feature extraction and spatial analysis within a single framework, enhancing efficiency and accessibility.

PRISM is an innovative Python package designed for end‐to‐end analysis of high‐dimensional multiplexed tissue microarray (TMA) data from spatial proteomic platforms such as CODEX and CosMx SMI, integrating interactive image processing, single‐cell analysis and spatial omics workflows within a unified framework.^98^ Built on the SpatialData standard, it ensures interoperability with tools like Scanpy^99^ and Squidpy^100^ while leveraging GPU acceleration for scalable computation. Key features include modular TMA dearraying, customisable Cellpose‐based^101^ cell segmentation, iterative cell‐type annotation via unsupervised clustering and spatial neighbourhood analysis, all accessible through an intuitive napari‐based graphical user interface (GUI) that enables real‐time validation of intermediate steps. By introducing AnnDataTrees to track hierarchical analysis checkpoints and supporting high‐plex datasets with parallel processing, PRISM bridges computational and translational research, facilitating the correlation of spatial protein expression with clinical outcomes in applications like cancer immunotherapy.

SPACEc (Structured Spatial Analysis for Codex Exploration) is a scalable, end‐to‐end Python framework designed for multiplexed imaging analysis, integrating tissue extraction, cell segmentation (via Mesmer^102^/CellPose^101^), data normalisation and spatial analysis with machine learning (ML) capabilities.^103^ It supports GPU‐accelerated workflows for large datasets (8–40 GB per image), combining unsupervised clustering (Leiden/Louvain) and supervised annotation (Support Vector Machine/STELLAR^104^) to identify cell types and spatial patterns. The modular pipeline accommodates data from CODEX, multiplex ion beam imaging (MIBI) and imaging mass cytometry (IMC) platforms, integrates with the scverse ecosystem and enables interactive visualisation via TissUUmaps.^105^ Demonstrated in healthy and inflamed tonsil studies, SPACEc efficiently uncovers cellular neighbourhoods (CN), CN interfaces and cell–cell interactions, bridging high‐dimensional imaging data to biological insights.

SPEX (Spatial Expression Explorer) is a modular, web‐based platform designed for end‐to‐end analysis of spatial omics data.^106^ It supports a wide range of imaging modalities, including IMC, MIBI and spatial transcriptomics technologies like MERFISH. It integrates image processing, single‐cell segmentation, clustering and spatial analysis tools into a user‐friendly interface that requires no advanced coding skills. SPEX supports diverse imaging modalities and data formats, enabling researchers to build customised pipelines for efficient data processing and interactive visualisation.

SpatialOne is an end‐to‐end, low‐code pipeline designed to simplify the analysis of 10X Visium spatial transcriptomics data, integrating a suite of specialised tools for various tasks to achieve near single‐cell resolution insights into tissue architecture and gene expression.^107^ In its upstream analysis, SpatialOne employs Cellpose^101^ and HoverNet^108^ for cell segmentation, generating spatial coordinates, masks and morphological features, while utilising Cell2Location^52^ or CARD^51^ for cell deconvolution to estimate cell types and proportions within Visium spots by matching transcript counts to single‐cell RNA expression profiles. For spatial structure analysis, it leverages Moran's I for identifying spatial clusters, neighbourhood enrichment for co‐occurrence patterns, BANKSY^86^ for detecting spatial domains, and SpatialDE^70^ for detecting SVGs, alongside comparative and regional analyses using SpaceRanger's clustering and two‐sided *Z*‐tests for cell infiltration quantification. Outputs are saved in CSV and AnnData formats, with spatial structure analyses documented in HTML reports and visualised interactively through TissUUmaps,^105^ enabling researchers to explore segmentation masks, inferred cell types and gene expression layers with ease, all packaged within a reproducible Docker container for scalability across diverse computational environments.

ENACT (End‐to‐End Analysis of Visium High Definition Data) is a self‐contained pipeline tailored for Visium HD platform, integrating advanced cell segmentation and bin‐to‐cell transcript assignment to achieve accurate single‐cell resolution analysis.^109^ It employs tools like Stardist^110^ and ML‐based methods (Sargent,^111^ CellAssign^112^ or CellTypist^113^) to enhance transcript mapping precision in complex, densely packed tissues. Validated across diverse synthetic and real datasets, including human and mouse tissue samples, ENACT demonstrates scalability and robustness, making it a valuable open‐source tool for spatially resolved transcriptomics studies. The pipeline's compatibility with downstream tools like SquidPy further supports comprehensive spatial analyses of cellular interactions and tissue architecture.

## 4 CTR RESEARCH USING SPATIAL OMICS TECHNOLOGIES

### 4.1 Chemotherapy

*Chemotherapy*, despite its widespread use in cancer treatment, is often limited by therapy resistance, which reduces its long‐term efficacy.^4^, ^115^ Traditional methods fail to adequately capture the spatial heterogeneity and dynamic interactions within the TME that drive resistance. By providing high‐resolution views of tissue heterogeneity and cellular interactions, spatial omics facilitates the identification of potential therapeutic targets to overcome chemo‐resistance.

Kulasinghe et al.^116^ utilised GeoMx DSP with a 68 antibodies panel to examine 24 tissue specimens from patients diagnosed with triple‐negative breast cancer (TNBC). These patients received 5‐fluorouracil, epirubicin and cyclophosphamide adjuvant chemotherapy, among whom nine experienced recurrences, while 15 did not. Differential expression analysis using the Limma package, complemented by sparse partial least squares‐discriminant analysis, identified protein markers that correlated with chemotherapy response. Within the stromal compartment, ER‐alpha expression positively correlated with chemotherapy response, whereas 4‐1BB and MART1 exhibited a negative correlation. Conversely, in the tumour compartment, GZMA, STING and fibronectin displayed a positive correlation with chemotherapy response, while CD80 showed a negative correlation. Furthermore, ER‐alpha expression in the stromal area was positively associated with extended overall survival (OS), contrasting with MART1, which was negatively associated with OS. In the tumour compartment, PD‐L1, FOXP3, GITR and SMA were positively correlated with extended OS, whereas EPCAM and CD95 were negatively associated with OS.

Similarly, Donati et al.^117^ utilised GeoMx DSP on 24 patients of TNBC treated with neoadjuvant chemotherapy of anthracycline and taxanes. They profiled over 200 regions of interest (ROI) using the GeoMx Cancer Transcriptome Atlas panel, which includes 1800 genes related to tumour biology and the TME. Using GeoMx DSP analysis suite, they observed that patients exhibiting a pathological complete response displayed enhanced IFN‐signalling and immune cell infiltration within tumour regions compared with those with progressive or no response. However, only modest differences were observed in the stroma, indicating a spatial heterogeneity of the immune infiltrate. These spatially‐defined molecular characterisation highlighted the potential of spatial omics in unravelling complex interactions within the TME contributing to chemotherapy resistance.

Spatial transcriptomics analysis also facilitated discovery of novel mechanisms of chemotherapy resistance. For example, Agostini et al.^118^ employed GeoMx DSP whole transcriptomics analysis to investigate 43 intraductal papillary mucinous neoplasms (IPMN) of the pancreas, comprising 12 low‐grade and 31 high‐grade (HG) samples, treated with gemcitabine and nab‐paclitaxel. Through differential expression analysis followed by gene set enrichment analysis (GSEA), they identified mucin‐specific O‐glycosylation as the most enriched pathway in HG‐IPMN, with GCNT3 markedly up‐regulated. Subsequent in vitro and in vivo experiments using both human and mouse organoids validated the function of GCNT3 in driving chemo‐resistance and the efficacy of GCNT3 inhibition by talniflumate.

Utilising 10X Visium combined with single‐nucleus RNA‐seq on 10 ovarian clear cell carcinoma (OCCC) samples (five chemo‐resistant and five chemo‐sensitive), Mori et al.^119^ identified that chemo‐resistant cancer cells with elevated HIF‐1α activity localised in cancer‐associated fibroblast (CAF)‐rich regions through a platelet‐derived growth factor (PDGF)‐mediated paracrine signalling. Functional validation using in vitro co‐culture systems and in vivo xenograft models further demonstrated that inhibiting PDGF signalling with ripretinib, in combination with carboplatin, effectively reduced tumour growth and overcame chemoresistance.

Cheng et al.^120^, ^121^ analysed 10X Visium data on four colorectal cancer (CRC) samples, including two liver metastases, to map spatial expression of CDKN2A and its interactions within the TME. Through integration with scRNA‐seq, scATAC‐seq and TCGA multi‐omics analysis, the study demonstrated that CDKN2A overexpression is linked to increased immune infiltration, altered copper metabolism and elevated glycolysis in CRC epithelial cells. This enhances immune suppression and promotes tumour invasion via the Wnt pathway and epithelial–mesenchymal transition (EMT)‐related mechanisms. These spatially resolved insights highlighted CDKN2A as a promising biomarker for predicting CRC resistance to chemotherapy (5‐fluorouracil) and radiotherapy, with significant implications for therapy‐resistant tumour niches.

Shiau et al.^122^ utilised CosMx SMI on 13 pancreatic ductal adenocarcinoma (PDAC) samples, with six receiving neoadjuvant therapy comprising 8–12 cycles of FOLFIRINOX chemotherapy, followed by fractionated radiotherapy (30–50 Gy EQD2) with concurrent 5‐fluorouracil or capecitabine, and the rest seven without such treatments. The authors developed the SCOTIA (Spatially Constrained Optimal Transport Interaction Analysis) algorithm, enabling the detection of spatially constrained ligand–receptor interactions within the TME. Through SCOTIA, they uncovered therapy‐associated remodelling, particularly an enrichment of IL‐6 family signalling pathway between CAFs and malignant cells in treated tumours. These findings provide spatial insights into molecular interactions that may drive chemoresistance in pancreatic cancer.

Lemaitre et al.^123^ utilised Akoya CODEX on 108 human hepatocellular carcinoma (HCC) patients samples, which included 53 primary untreated samples and 55 residual samples following transarterial chemoembolisation. The study complemented this with GeoMx DSP analysis on murine models, focusing on minimal residual disease (MRD). By employing these spatial profiling techniques, the study suggested that PD‐L1^+^ macrophages interacted with stem‐like tumour cells within spatially distinct microenvironments, leading to T‐cell exhaustion and persistence of residual cells. This spatial understanding of tumour–immune cell interactions underscores the potential of TGFβ1 and PD‐L1 pathway inhibition to prevent HCC recurrence by disrupting immune‐evasion mechanisms in MRD.

Kats et al.^124^ utilised 10X Visium on 13 patient SHH‐medulloblastoma samples (eight SHH‐TP53mut and five non‐chromothriptic sporadic SHH‐TP53wt), along with 11 patient‐derived xenograft (PDX) models treated with carbon ion radiation and PARP inhibitors. The study integrated FISH validation on adjacent tissue sections and previously generated matched single‐cell DNA/RNA sequencing data.^125^ Their findings indicated that chromothriptic medulloblastomas exhibit pronounced spatial intra‐tumour heterogeneity, characterised by increased proliferative and stemness signatures, alongside reduced immune infiltration. These findings highlight how distinct spatial clonal architectures can drive therapy resistance and relapse.

Zhou et al.^126^, ^127^ re‐analysed eight 10X Visium CRC samples from patients treated with oxaliplatin‐based chemotherapy. They found that THBS2⁺ CAFs were spatially proximate to oxaliplatin‐resistant malignant cells and actively interacted with them via collagen, underscoring their pivotal role in mediating chemoresistance. Furthermore, by incorporating pan‐cancer analyses using TCGA bulk RNA‐seq, pan‐cancer protein data, pan‐cancer cell line expression data, pan‐cancer scRNA‐seq data and public spatial transcriptomics data of ovarian cancer (OC; 10X official data), BRCA (10X official data), PAAD^128^ and HNSC,^129^ the authors demonstrated that THBS2⁺ CAFs promote EMT and drive oxaliplatin resistance via COL8A1‐mediated activation of the PI3K–AKT pathway.

Kiviaho et al.^130^ utilised 10X Visium on 80 fresh‐frozen tissue sections from 56 prostatectomy samples—including benign prostatic hyperplasia, treatment‐naïve, neoadjuvant‐treated (anti‐androgen and chemical castration therapy) and castration‐resistant prostate cancers (CRPC). Through unsupervised clustering and integration with scRNA‐seq using non‐negative matrix factorisation, they delineated distinct spatial cell state regions, referred to as single‐cell mapping‐derived (SCM) regions, by identifying unique sets of differentially expressed, region‐specific marker genes across spatial spots. One SCM region, characterised by the overexpression of recently proposed club cell markers (MMP7, PIGR, CP and LTF), was consequently designated as the ‘Club region’.^131^ Notably, the Club region exhibited elevated inflammatory and senescence‐associated secretory phenotype gene signatures, and a strong spatial correlation was observed between club‐like cell‐rich areas and regions with high polymorphonuclear myeloid‐derived suppressor cell activity.

Carbone et al.^132^ employed the Stereo‐seq OMNI spatial transcriptomics platform on four tissue macro array sections from syngeneic orthotopic PDAC mouse models treated with the antibody–IL2 fusion protein (L19–IL2), administered either as monotherapy or in combination with FOLFOX. By integrating RNA‐seq, immunohistochemistry (IHC) and immunofluorescence (IF) data, the study demonstrated that L19–IL2 treatment spatially orchestrates a robust influx of cytotoxic CD8⁺ T cells and NK cells into the tumour core, effectively converting a ‘cold’ TME into a ‘hot’ one and potentially mitigating resistance to conventional cancer therapies. The limited sample size invites further clinical validation.

### 4.2 Endocrine therapy

*Endocrine therapy* is a cornerstone treatment for hormone‐dependent cancers, such as certain types of breast and prostate cancer.^133^, ^134^ Despite its effectiveness, many patients develop resistance, posing a significant challenge and underscoring the need to understand the underlying mechanisms of CTR.^135^, ^136^

Zhang et al.^137^ employed BGI Stereo‐seq to analyse four ER^+^HER2^–^ breast cancer samples (two endocrine‐sensitive and two endocrine‐resistant) from patients undergoing endocrine therapy post‐surgery and chemotherapy. Combining scRNA‐seq, this study revealed a reduced abundance of tumour‐infiltrating T cells in resistant tumours. Further deconvolution and pathway scoring (using SPOTlight^138^ and ssGSEA^139^) analyses illustrated a correlation between endocrine resistance and down‐regulated innate immune signalling for cytosolic DNA sensing through a feedback loop between suppressed STING and activated AKT1 signalling. Combination therapy with STING agonists and AKT1 inhibitors, as proposed, may disrupt this loop and enhance immune responses, offering a promising approach to address endocrine resistance in ER^+^HER2^–^ breast cancer.

Romero et al.^140^ applied 10X Visium on two adjacent sections from 10‐week RPM (Rb1^−/−^; Trp53^−/−^; cMyc^+^) mouse prostate cancer models, building on Ku et al.’s^141^ findings that Rb1 and Trp53 loss promotes lineage plasticity and antiandrogen resistance. By integrating spatial transcriptomics with multiplexed IF, this study revealed spatially distinct neuroendocrine (NE) cell populations emerging from KRT8^+^ luminal progenitors, which transit into heterogeneous ASCL1^+^ NE prostate cancer (NEPC) regions resistant to androgen receptor inhibitors. These NE populations were shown to be spatially dependent on the TME, offering potential spatial markers for therapy resistance. Such findings highlight how spatially organised cell states within the TME contribute to CTR, particularly in CRPC.

### 4.3 Targeted thearpy

Targeted therapy, designed to interfere with specific molecular targets involved in tumour progression, is challenged by tumour cell heterogeneity and resistance mechanisms.^142^, ^143^ Spatial omics technologies started to elucidate key mechanisms of resistance of targeted therapy across multiple cancer types.

Combining 10X Visium and scRNA‐seq, Jussila et al.^144^ investigated the transition of basal cell carcinoma (BCC) to squamous cell carcinoma (SCC), referred to as basal to squamous transition (BST), in a patient with Gorlin syndrome (nevoid BCC syndrome) under treatment of Smoothened inhibitor (SMO^i^). Tumour regions with distinctive gene expression patterns were identified to be correlated with BCC and SCC phenotypes. Specifically, regions manifesting SCC‐like features showed high levels of LY6D, a marker of squamous differentiation, and low levels of GLI1, a gene typically active in Hedgehog pathway‐driven BCCs. This differential expression across the tumour landscape provided a clear spatial map of BST under continuous SMO^i^ therapy leading to resistance. Further verification in more patient samples is needed to confirm if this is a general mechanism.

Li et al.^145^ utilised Akoya CODEX (*n* = 4) and 10X Visium (n = 2) on BCC samples to explore tumour–immune interactions under SMO^i^ therapy. By integrating spatial omics with scRNA‐seq and scATAC‐seq data, the study identifies a basal‐to‐inflammatory transition within a TREM1 myeloid cell‐enriched niche. This spatially confined inflammatory microenvironment, marked by IL1 and OSM signalling activating NF‐κB in tumour epithelial cells, fosters a drug‐resistant niche closely linked to SMO^i^ resistance.

Using 10X Visium and IHC data of human glioblastoma from the Spatial Omics Resource of Cancer database^146^ and STOmicsDB,^147^ Zhang et al.^148^ showed that TNFα expression is lower in tumour region of high expression of cancer stem cell markers CD133 and SOX2, compared with regions without stemness markers. By integrating RNA sequencing, mass spectrometry and in vitro/in vivo experiments, the study revealed that low TNFα levels in these regions promoted glioma stem cell self‐renewal via Vasorin‐mediated glycolysis, contributing to therapy resistance and highlighting the potential of targeting both TNFα and Vasorin pathways as a potential therapeutic strategy.

Izumi et al.^149^ employed 10X Visium on samples from EGFR^TL^/CCSP‐rtTA mouse model mimicking human lung cancer with EGFR‐mutant. Comparing untreated, short‐term EGFR‐TKI treatment reflecting a drug‐tolerant persister state, and recurrence state after prolonged treatment, this study identified spatial heterogeneity in expression of apoptosis‐related genes, particularly BCL2L1, in tumour cells. Furthermore, genetic ablation and pharmacological inhibition of BCL2L1/BCL‐XL mitigated the onset of therapy resistance.

Li et al.^150^ utilised 10X Visium on 10 samples of EGFR‐mutant lung adenocarcinoma (LUAD), including two never‐transformed LUAD samples (LUAD‐NT), three LUAD samples collected before transformation (LUAD‐BT), three small cell lung cancer samples after transformation (SCLC‐AT) and two primary SCLC samples (SCLC‐P). Some of these samples underwent transformation to SCLC after developing resistance to EGFR‐TKI therapy.^150^ This spatial data were integrated with bulk RNA sequencing and multiplex immunofluorescence (mIF) to reveal spatially distinct transcriptomic shifts during this transformation. Specifically, down‐regulation of HDAC10 in tumour cells and continued activity of FGF and VEGF signalling pathways in transformed SCLC highlighted tumour‐intrinsic changes as the primary drivers of resistance, rather than alterations of the TME.

Wang et al.^151^ applied MALDI–Fourier transform ion cyclotron resonance imaging mass spectrometry (MALDI–FT–ICR IMS) to analyse spatial metabolome of 42 pre‐treatment biopsy samples from patients with HER2‐positive advanced gastric cancer with trastuzumab therapy. The metabolic heterogeneity of individual patients was quantified using Simpson's diversity index, revealing a significantly correlation between high metabolic heterogeneity and increased sensitivity to trastuzumab. With K‐means clustering algorithm, nine distinct tumour subpopulations were identified. Two of these subpopulations were associated with a favourable prognosis and enhanced sensitivity to trastuzumab, while one subpopulation was linked to poor prognosis and resistance to trastuzumab.

Bouchard et al.^152^ developed a spatial cell–cell co‐localisation framework using mIF‐based spatial proteomics via the PhenoCycler platform. This framework was applied to in vitro assembloids consisting of LUAD patient‐derived organoids co‐cultured with regionally distinct CAFs, as well as matched clinical specimens from patients undergoing EGFR‐targeted erlotinib treatment. By employing quantitative analysis based on the colocation quotient and spatial permutation testing, they identified distinct spatial reorganisation patterns, notably a pronounced segregation between cancer cells and fibroblasts, which may provide mechanistic insights into the spatial reorganisation associated with drug resistance.

Rubinstein et al.^153^ utilised 10X Visium on two PDX models of BRAF‐mutant melanoma (WM4007 and WM4237) treated with targeted therapy (dabrafenib and trametinib). The study analysed multiple time points (T0, T1, T2, T3 and T4) during the treatment course, with one untreated control for each model. Through spatial transcriptomics and deep learning analysis of histopathologic slides, the study identified conserved spatial patterns, including central‐to‐peripheral gradients in gene expression and cell state transitions, highlighting the role of spatial organisation in treatment resistance.

Hong et al.^154^ utilised 10X Visium on 3 prostate cancer samples (including acinar cell carcinoma and adenocarcinoma) treated with 177Lu‐ and 225Ac‐labelled *radiopharmaceutical therapy*. They demonstrated that spatial transcriptomics spots exhibiting high prostate‐specific membrane antigen density correlate with increased absorbed dose and reduced cell survival in tumour cell‐rich regions. In contrast, tumour cell‐depleted and hypoxic areas exhibited higher resistance, particularly under 177Lu‐based therapy, whereas 225Ac displayed enhanced efficacy in overcoming these microenvironmental barriers.

Together, these studies illustrate the potential that generation of spatially resolved maps of tumour cell and microenvironment heterogeneity may facilitate the development of more precise targeted therapeutic strategies to overcome resistance and improve patient outcomes.

### 4.4 Immunotherapy

*Immunotherapy* has revolutionised cancer treatment by harnessing the body's immune system to combat the disease. However, it encounters significant challenges due to resistance, resulting in only a limited fraction of patients deriving benefit from these therapies.^155^, ^156^ Spatial omics may provide a comprehensive understanding of how tumour heterogeneity, immune cell infiltration and tumour–stroma interactions shape resistance to immunotherapy.

#### 4.4.1 Tumour heterogeneity

Understanding tumour heterogeneity is crucial for addressing resistance to immunotherapy. Spatial omics technologies have unveiled intricate patterns of gene expression and cellular distribution within tumours that contribute to therapeutic outcomes.

Sun et al.^157^ analysed 10X Visium data of 6 LUAD samples and identified that overexpression of EGLN3 in cancerous and hypoxic regions was positively correlated with expression of DNA damage response and TGF‐β pathway genes. By integrating data from multiple sources, their analysis suggested that EGLN3 could be a potential biomarker for poor responses to both immunotherapy and chemotherapy. Lozar et al.^158^ employed GeoMx DSP to analyse samples from 10 patients with head and neck SCCs (HNSCC) comprising three responders and seven non‐responders to pembrolizumab‐based immunotherapy. Profiling of 51 ROI identified transcriptional differences—most notably IDO1, HLA‐F, S100A8 and S100A9—associated with therapeutic outcomes. High CK17 expression was correlated with poorer response, shorter time to treatment failure and progression‐free survival (PFS), highlighting intra‐tumoural heterogeneity affecting therapy response.

Xia et al.^159^ utilised 10X Visium on two OCCC samples, integrating these data with scRNA‐seq and IHC from additional patients, including those treated with combination therapy comprising VEGF inhibitors and anti‐PD‐1 antibodies, to interrogate the TME. Employing a Seurat‑based clustering approach and ligand–receptor interaction analysis (CellChat v2^160^), they identified distinct spatial niches (epithelial‑, stromal‑, endothelial‑ and immune‑rich regions) and demonstrated the co‐localisation of key molecules, such as CD47 and THBS1, which may contribute to immunosuppression. Although these findings enhance our understanding of the spatial heterogeneity underlying therapy resistance in OCCC, the limited sample size invites further validation to confirm broader clinical relevance.

Jhaveri et al.^161^ utilised CODEX with a 101‐antibody panel to analyse one primary HNSCC tissue sample from a patient treated with pembrolizumab, exhibiting a partial response in measurable disease, supplemented by retrospective samples from four additional patients treated with immune checkpoint inhibitors (ICIs). Through unsupervised clustering and CN analysis, they identified six distinct spatial neighbourhoods enriched with immune subsets and four metabolically unique tumour regions. These spatial findings highlighted significant intra‐tumoural heterogeneity, revealing a dichotomy between immune activation‐induced apoptosis and tumour progression within the same sample, thus providing novel insights into the TME architecture. By integrating single‐cell spatial proteomics data with functional states, the study advanced the understanding of CTR, particularly to ICIs, by identifying potential spatial biomarkers such as G6PD and MMP9 overexpression that may predict variable clinical responses, thereby offering a framework for stratifying patients and guiding immunotherapy optimisation.

#### 4.4.2 Immune cell infiltration

The extent and nature of immune cell infiltration within tumours are critical determinants of immunotherapy success. Spatial omics has shed light on how immune cells are distributed and interact within the TME.

Larroquette et al.^162^ used GeoMx DSP on 16 tumour samples from non‐small cell lung cancer (NSCLC) patients treated with PD‐1/PD‐L1 inhibitors. They demonstrated that elevated intra‐tumoural CD163^+^ macrophage infiltration was correlated with shorter PFS and OS. Furthermore, tumours with substantial macrophage infiltration showed elevated expression of genes related to interferon‐γ signalling pathway and responded better to treatment. These findings underscored a critical role of macrophages in modulating the immune microenvironment of NSCLC. Similarly, Berrell et al.^163^ utilised GeoMx DSP and Akoya PhenoCycler to perform spatial transcriptomics and proteomics on 25 tissue cores from 12 patients with HNSCC undergoing immunotherapy (pembrolizumab or nivolumab), including 8 cores from four unresponsive patients, 5 cores from two responsive patients and 12 cores from six naive patients. They identified differential expression of hypoxia and complement system genes in non‐responders, while immune cell, particularly CD8^+^ T cell, infiltration was higher in responders, suggesting that spatially resolved tumour–immune interactions may contribute to therapy resistance in HNSCC. Hu et al.^164^, ^165^ re‐analysed spatial transcriptomics (10X) data from seven liver HCC (LIHC) samples, comprising five ICB non‐responders and two responders. Through multi‐omics integration and a ML framework, they developed a mitochondrial cell death index (MCDI) to evaluate therapy responses, particularly to ICB and sorafenib. This spatially resolved analysis demonstrated a strong correlation between MCDI levels, immune infiltration and therapeutic resistance in LIHC, offering a predictive marker for personalised therapy response and shedding light on mechanisms underlying therapy resistance.

Quek et al.^166^ utilised CODEX on to analyse tumour samples from five patients with metastatic melanoma who were treated with anti‐PD‐1 and anti‐CTLA‐4 combination immunotherapy. Through the integration of scRNA‐seq and CITE‐seq data with spatial imaging, the study identified an ‘immune‐striving’ TME characterised by peri‐tumour lymphoid aggregates and low T cell infiltration in the tumour core, which was associated with resistance to immunotherapy. The enrichment of B cell signatures in lymphoid aggregates was found to be associated with better survival outcomes.

Monkman et al.^167^ utilised CODEX on 35 pre‐treatment TMA samples from NSCLC patients treated with PD1 axis ICIs to investigate spatial immune‐tumour interactions. Through an analytical pipeline involving unsupervised clustering (Phenograph), spatial interaction analysis (Scimap), CN detection and SpatialScore metrics, the study revealed that regulatory T cells (Tregs) were enriched in stromal and peripheral tumour‐margin regions of non‐responding patients, with increased proximity to monocytes (*p* = .009) and CD8⁺ T cells (*p* = .009), while macrophages were more frequently associated with HLADR⁺ tumour cells (*p* = .01) in responders. These spatial findings suggest that Treg‐mediated immunosuppression, potentially driving an M2 macrophage phenotype, contributes to ICI resistance, whereas macrophage proximity to tumour cells and effector CD4⁺ T cell enrichment in mixed tumour neighbourhoods correlate with response, offering insights into overcoming resistance to cancer therapy.

#### 4.4.3 Tertiary lymphoid structures

Tertiary lymphoid structures (TLSs) are organised immune aggregates in chronically inflamed tissues, including tumours. Comprising B‐cell follicles (CD20⁺), T cells (CD3⁺) and dendritic cells, TLSs enhance anti‐tumour immunity by facilitating antigen presentation and lymphocyte activation.^168^ In cancer, TLSs correlate with improved responses to immunotherapy (e.g., checkpoint inhibitors), chemotherapy and surgery, with their maturity and spatial location (intra‐tumoural/peri‐tumoural) serving as prognostic biomarkers.

In HG serous ovarian cancer (HGSOC), MacFawn et al.^169^ applied the GeoMx DSP to analyse tumour samples from seven ovarian, four omental and two Fallopian tube HGSOC cases. Their work identified a prognostic 20‐gene TLS‐DSP signature and revealed significant heterogeneity in the maturity and spatial distribution of TLSs across tumour sites, with implications for therapy resistance. TLS within Fallopian tube and omental tumours exhibited higher maturity—marked by germinal centres and B cell activation—and correlated with improved survival, suggesting enhanced immunotherapy response. In contrast, ovarian TLS were less frequent and functionally impaired, a phenotype linked to immunosuppressive cancer‐associated mesenchymal stem cells (CA‐MSCs) that spatially co‐localised with TLS and suppressed B cell activity.

In CRC liver metastasis (CRLM), Zhang et al.^170^ demonstrate that TLSs in the peri‐tumoural region (within 2 mm of the tumour margin) correlate with improved survival outcomes after surgical resection, particularly when TLSs are mature (FL2 follicles). Using Stereo‐seq on 10 CRLM samples (6 TLS+, 4 TLS−), the authors identified 154 TLS‐like structures and revealed that TLS+ regions are enriched with IgG+ plasma cells. These plasma cells spatially co‐localise with CCL19‐producing fibroblasts, which in turn induce macrophage‐mediated tumour cell apoptosis. These findings highlight TLS maturity and spatial location as prognostic biomarkers for CRLM, as TLS+ tumours exhibit enhanced anti‐tumour IgG responses and reduced resistance to immunotherapy.

In NSCLC, Peyraud et al.^171^ demonstrate that mature TLSs (mTLSs), identified by CD23⁺ follicular dendritic cells and located intra‐tumourally, correlate with improved responses to ICIs, independently of PD‐L1 expression. Using spatial transcriptomics (GeoMx), the study reveals that primary resistance to ICIs in mTLS‐positive tumours is driven by two distinct CAFs subsets: FAP⁺αSMA⁺ CAFs, linked to CD8⁺ T cell exhaustion and inflammatory pathways, and MYH11⁺αSMA⁺ CAFs, which promote immunosuppression via CD4⁺ Treg infiltration. Spatial transcriptomics not only enhanced the precision of TLS detection but also uncovered stromal CAF signatures associated with immune exclusion and exhaustion, highlighting their role in shaping the TME. These findings underscore mTLSs as robust predictive biomarkers for ICIs and identify CAFs as potential therapeutic targets to overcome resistance of ICIs in NSCLC.

In a study of NSCLC patients treated with neoadjuvant PD‐1 inhibitors combined with chemotherapy, Yan et al.^172^ utilised spatial transcriptomic on 14 pre‐treatment (GeoMx) and 17 post‐treatment (Visium) samples. Their findings demonstrated that activated TLSs, marked by germinal centre B cells and follicular helper T cells, localised distal to tumour regions. These structures correlated with reduced residual tumour burden and enhanced survival. Conversely, hypoxic TLSs, enriched in glycolysis and hypoxia signatures, clustered near residual tumour cells in non‐responders, correlating with poor outcomes. and TLS maturation stages (early lymphoid aggregates, activated, declining, late) are influenced by the TME, with hypoxia suppressing TLS activation through Treg enrichment and CD4 effector T cell inhibition. Additionally, spatial mapping identified immunosuppressive barriers at tumour boundaries, formed by COL11A1+ CAFs and SPP1+ macrophages, which physically block T cell infiltration.

In TNBC, Wang et al.^173^ applied spatial transcriptomics to 92 patients, identified a 30‐gene TLS signature enriched in B‐cell markers (CD79A, CD20) and lymphoid priming genes (CXCL13, CCL19). This signature predicts improved survival and response to immunotherapy in TNBC and other cancers. Spatial analysis revealed TLS are predominantly intra‐tumoural or stromal, with mTLS (marked by POU2AF1) correlating with ‘fully inflamed’ immune microenvironments and favourable outcomes. By integrating spatial transcriptomics with bulk RNA‐seq, the study identified nine spatial archetypes, including TLS‐rich SA4 (characterised by immune activation and angiogenesis) and immunosuppressive SA8 (marked by NECTIN4 overexpression). These archetypes stratify patients based on therapeutic vulnerabilities, highlighting TLS as a critical biomarker for precision oncology in TNBC.

In muscle‐invasive bladder cancer (MIBC), Lin et al.^174^ utilised Visium on 7 paraffin‐embedded MIBC samples (three TLS‐positive, two TLS‐negative, one lymph node metastasis). Their analysis identified stromal TLSs, characterised by CXCL13+ T cell and NR4A2+ B cell interactions along with IGHG secreting plasma cells, which correlated with improved prognosis and enhanced antitumour immunity. Through integration of spatial transcriptomics, scRNAseq and IHC, the authors identified mTLS as spatial biomarkers linked to favourable immunotherapy responses, with CXCL13 emerging as a critical driver of TLS assembly. A TLS‐specific gene signature (RMYY‐TLS), derived from spatial transcriptomics data, demonstrated prognostic value across multiple cancer types.

Molina‐Alejandre et al.^175^ utilised 10X Visium on two post‐treatment samples from patients with HLA class I‐deficient NSCLC undergoing perioperative chemoimmunotherapy (ChIO), through a neoadjuvant regimen combining nivolumab, paclitaxel and carboplatin, followed by adjuvant nivolumab monotherapy. Through k‐means clustering, the authors identified significant immune infiltration patterns, including the presence of TLSs and activated T cells, specifically in tumours with a complete pathological response. These findings elucidate a potential mechanism by which TLS and cytotoxic T‐cell responses might contribute to overcoming immune escape in HLA‐deficient tumours, offering new insights into ChIO resistance.

#### 4.4.4 Tumour–stroma interactions

Interactions between tumour cells and surrounding stromal cells, including CAFs and immune cells, play a pivotal role in cancer progression and resistance to therapies.

Davidson et al.^176^ used 10X Visium and multiplex immune staining to identify the spatial proximity of mesenchymal‐like clear cell renal cell carcinoma (ccRCC) cells and myofibroblastic CAFs at the interface of normal adjacent tissue. Combined with scRNA‐seq and public dataset analysis, their data revealed a correlation between mesenchymal‐like ccRCC cells and myCAFs, both enriched in metastatic sites and associated with poor patient survival. These findings illustrated that epithelial–mesenchymal plasticity of ccRCC cancer cells and their interaction with myCAFs may be linked with resistance to immune checkpoint therapies.

Di Modugno et al.^177^ utilised GeoMx DSP to analyse two NSCLC patients treated with immune checkpoint blockade (ICB) therapies (nivolumab and pembrolizumab), one responder and one non‐responder. By spatially profiling 1800 transcripts and using custom probes for hMENA splice variants, along with staining immune, stromal and epithelial compartments, they identified that the hMENA^11a^ isoform is up‐regulated in the tumour compartment of ICB responders, while FN1 is overexpressed in non‐responders. These findings highlighted the potential role of hMENA isoforms in regulating TLSs and provided potential biomarkers for ICB therapy resistance in NSCLC.

Zhang et al. re‐analysed six 10X Visium ccRCC frozen sections from the GSE175540^178^ dataset, integrating scRNA‐seq and mIF data from patients treated with ICIs. Their study demonstrated that SPP1‐secreting tumour cells interacts with CD44‐expressing exhausted CD8⁺ T cells in situ, thereby reshaping the immune microenvironment and driving primary resistance.^179^

Naei et al.^180^ employed a 53‐plex antibody panel on the PhenoCycler‐Fusion platform to analyse FFPE samples from 15 HNSCC patients undergoing anti‐PD‐1 immunotherapy. By integrating in situ proximity ligation assays (isPLA; Navinci Diagnostics) to map the PD‐1/PD‐L1 interaction landscape, they identified macrophage–tumour cell barriers linked to immunotherapy resistance, as well as B‐ and T‐cell aggregates near tumour boundaries in responders.^180^

#### 4.4.5 Multiple resistance mechanisms

Zhang et al.^181^ employed 10X Visium on 12 HCC samples from patients receiving neoadjuvant cabozantinib and nivolumab, of which five exhibited a pathologic response. Their analysis revealed that patients with significant pathologic response followed by early recurrence displayed pronounced tumour heterogeneity, with an immune‐poor region marked by HCC–CAF interactions and cancer stem cell marker expression, potentially facilitating immune evasion and recurrence. Additionally, responding HCC tumours showed increased immune cell infiltration and an enrichment of CAFs expressing pro‐inflammatory genes, alongside enhanced ECM remodelling and tumour–stroma interactions, which may promote immune‐driven tumour regression.

Wu et al.^182^ applied 10X Visium on 4 TNBC samples, showing that KLF5 expression co‐localised with basal markers (KRT5, KRT14, KRT17) in tumour regions, while areas with reduced KLF5 expression were enriched with CD4^+^ and CD8^+^ T cells. This inverse pattern indicates that intra‐tumoural heterogeneity in KLF5 expression may influence immune cell infiltration and potentially drive resistance to immunotherapy.

Salachan et al.^183^ conducted 10X Visium on primary prostate tumours from three metastatic prostate cancer patients undergoing palliative TURP, including one case of CRPC and two NEPC cases. In NEPC samples, canonical immune checkpoint genes PDCD1, CD274 and PDCD1LG2 were absent, while CD276 was uniformly expressed across tumour regions. Dysregulated immune genes associated with disease aggressiveness were identified in malignant NEPC subtypes, underscoring the role of tumour heterogeneity in therapy resistance. Additionally, CAF‐pro‐tumour macrophage interactions were evident with SpaCE analysis, suggesting CAF involvement in modulating the immune microenvironment.

Fan et al.^184^ utilised Stereo‐seq and mass spectrometry‐based spatial proteomics to analyse 15 cervical SCC (CSCC) samples. Their spatial omics findings showed that epithelial–cytokeratin tumours were spatially separated from immune cell infiltrates, while epithelial‐immune tumours were closely associated with nearby immune cells. This suggested that this spatial arrangement may impact immune checkpoint therapy resistance. Additionally, immunosuppressive CAFs with active TGFβ signalling were specifically located around epithelial–cytokeratin tumours. Using a modified CellChat algorithm, the study identified that TGFβ‐mediated ligand–receptor interactions activate adjacent immunosuppressive TGFBR2‐expressing CAFs, potentially contributing to immune checkpoint therapy resistance in CSCC.

Wang et al.^185^ analysed spatial transcriptomics data from 13 HCC samples (tumour, normal and peri‐tumoural regions) using the ST platform and additional data from 8 anti‐PD‐1‐treated HCC patients using 10X Visium. Their findings revealed a significant spatial co‐localisation of POSTN^+^ CAFs with SPP1^+^ macrophages, particularly in patients resistant to immunotherapy. These interactions were shown to obstruct CD8^+^ T cell infiltration into the tumour core, fostering an immunosuppressive microenvironment that contributes to therapy resistance.

Schaer et al.^186^ employed 10X Visium on two MC38 colon adenocarcinoma mouse samples treated with anti‐CD40 antibodies, revealing clusters of NRF2‐activated, tumour‐promoting TAMs in peri‐necrotic haemorrhagic tumour regions, resembling anti‐inflammatory erythrophagocytic macrophages. Functional assays and scRNA‐seq of TAMs in 3D culture spheroids further demonstrated that intra‐cellular heme‐NRF2 signalling activated cancer‐promoting features in TAMs, inducing tumour cell EMT and resistance to IFNγ and anti‐CD40 antibodies. These findings underscore the role of tumour heterogeneity in therapy resistance and suggest potential therapeutic targets within tumour–stroma interactions for enhancing immunotherapy efficacy.

Xun et al. analysed seven tumour samples across five cancer types—BRCA, SCC, ccRCC, CRC and OC—revealing a hypoxia‐driven ALCAM^high^ macrophage‐exhausted T cell axis localised predominantly at the tumour boundary.^178^, ^187^, ^188^, ^189^ By integrating bulk RNA‐seq, single‐cell RNA‐seq, this study underlined that hypoxia fosters immune resistance by inducing ALCAM expression in macrophages, which co‐localise with exhausted CD8+ T cells, upon ICB and hypoxia inhibition therapies.

Fan et al.^190^ utilised 10X Visium on six intra‐hepatic cholangiocarcinoma samples to investigate tumour–immune cell interactions in the context of immune resistance to cancer therapies. By integrating spatial and scRNA‐seq data, the authors identified a spatial co‐localisation of MARCO^+^ TAMs and CTSE^+^ tumour cells, which was linked to an immune‐suppressive TME and poorer prognosis. Further analysis suggested that this spatial co‐localisation facilitated immune escape through the LGALS9–CD44 signalling pathway.

Aung et al.^191^ utilised GeoMx DSP on 55 discovery and 45 validation samples of advanced melanoma (unresectable stage III or IV) treated with PD‐1‐based ICIs. Through a computational pipeline involving LASSO‐regularised logistic regression, it identified compartment‐specific gene signatures (S100B, CD68, CD45) that leverage spatial transcriptomic data to predict immunotherapy outcomes with higher accuracy than bulk RNA‐seq signatures. The study revealed that spatial transcriptomics captures compartment‐specific expression patterns, with the S100B signature achieving an AUC of 0.79 in validation, significantly surpassing pseudo‐bulk (AUC 0.70) and showing specificity through cross‐compartment testing (e.g., *p* = 9.6e−05 for S100B vs. CD68). This advancement aids CTR by offering a refined, spatially informed tool for stratifying melanoma patients likely to respond to ICIs, potentially reducing ineffective treatments and informing personalised therapeutic strategies.

Soupir et al.^192^ employed NanoString CosMx SMI to analyse 42 FFPE cores from 21 ccRCC patients, including both immunotherapy‐naïve and immunotherapy‐exposed cases. Their spatial transcriptomics and mIF analyses revealed a significant increase in the spatial coupling of integrin αV and collagen IV, along with an enrichment of EMT signalling in the TME.

**FIGURE 2:**
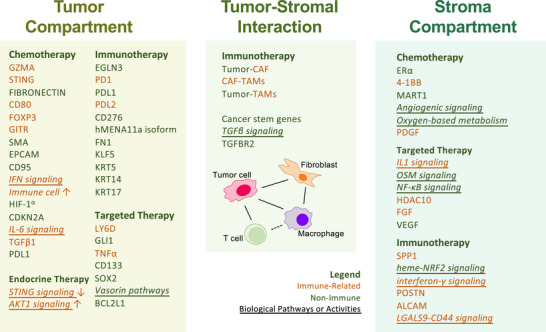
Molecular signatures driving CTR unveiled by spatial omics studies. This figure summarises key CTR‐associated molecular signatures compiled from spatial omics studies discussed in the preceding sections. Markers are categorised by tumour compartment, stromal compartment and tumour–stroma interactions, spanning chemotherapy, immunotherapy, targeted and endocrine therapy contexts. Immune‐related features are highlighted in orange, and biological pathways are shown in green italics.

By identifying localised biomarkers and resistance mechanisms (Figure 2), spatial omics reveals potential therapeutic targets that could enhance the efficacy of immunotherapies across different cancer types.

## 5 CONCLUSION AND OUTLOOK

In this review, we highlighted the transformative role of spatial omics in deciphering complex mechanisms underlying CTR. Unlike traditional bulk sequencing or single‐cell methods, which miss the spatial context, spatial omics allows researchers to pinpoint molecules driving resistance within specific tumour regions and explore how their locations affect treatment effects. As shown throughout this review, it has greatly advanced CTR research by revealing resistant niches, uncovering cell–cell interactions, identifying spatial biomarkers and shedding light on immune dynamics. Specifically, spatial omics identifies spatially restricted subclones and their microenvironmental dependencies that contribute to therapeutic resistance, maps ligand–receptor interactions underlying immune suppression or tumour survival, thereby revealing novel therapeutic targets, and improves the prediction of treatment outcomes by anchoring molecular signatures to their precise spatial contexts.

A primary *advantage* of spatial omics lies in its ability to unveil spatial heterogeneity and resistant niches within tumours. Tumours comprise heterogeneous subclones with distinct spatial distributions, shaped by local microenvironmental cues that mediate responses to therapeutic selective pressures. For instance, Mori et al.^119^ employed 10X Visium in OCCC to reveal chemo‐resistant cells with elevated HIF‐1α activity localised in CAF‐rich regions, driven by PDGF‐mediated signalling. Subsequent inhibition of this pathway restored chemosensitivity, underscoring spatial omics’ capacity to link molecular signatures to geographic locales. Similarly, Kats et al.^124^ applied 10X Visium to medulloblastoma, identifying chromothriptic tumours with spatially segregated proliferative and stemness signatures associated with reduced immune infiltration and therapy resistance. In CRC, Zhou et al.^126^ demonstrated that THBS2+ CAFs, spatially proximate to oxaliplatin‐resistant cells, promote EMT via collagen signalling, enhancing resistance. Romero et al.,^140^ studying prostate cancer, identified spatially distinct NE cell populations resistant to androgen receptor inhibitors, reliant on TME interactions. These findings collectively illustrate how spatial omics pinpoints resistant subclones and their microenvironmental dependencies, offering critical insights into tumour evolution under therapy.

Beyond heterogeneity, spatial omics provides unparalleled precision in mapping cell–cell interactions in situ. Resistance often emerges from dynamic exchanges among tumour, stromal and immune cells, with spatial organisation directly influencing therapeutic outcomes. Shiau et al.^122^ utilised CosMx SMI in PDAC to detect therapy‐induced IL‐6 family signalling between CAFs and malignant cells, a network potentially sustaining chemoresistance. In HCC, Lemaitre et al.^123^ combined CODEX and GeoMx DSP to show PD‐L1‐positive macrophages interacting with stem‐like tumour cells in residual disease, promoting T‐cell exhaustion and resistance persistence. Davidson et al.,^176^ in ccRCC, observed mesenchymal‐like tumour cells spatially adjacent to myofibroblastic CAFs at the tumour–normal interface, a pattern linked to immunotherapy resistance. Fan et al.,^184^ using Stereo‐seq in CSCC, found TGFβ‐mediated CAF activation near epithelial tumours, creating an immunosuppressive niche. These studies highlight spatial omics’ ability to delineate ligand–receptor networks and cellular crosstalk, identifying actionable therapeutic targets within the TME.

Another pivotal contribution of spatial omics is its capacity to identify spatial biomarkers associated with therapy response or resistance. By correlating molecular profiles with spatial locations and clinical outcomes, this approach uncovers predictive markers surpassing traditional molecular indicators. Kulasinghe et al.,^116^ in TNBC, used GeoMx DSP to identify stromal ER‐alpha and tumour‐specific proteins (e.g., GZMA, STING) as predictors of chemotherapy response and survival. Cheng et al.,^121^ in CRC, linked CDKN2A overexpression in immunosuppressive niches to resistance against chemotherapy and radiotherapy. Zhang et al.,^137^ employing Stereo‐seq in breast cancer, associated reduced T‐cell infiltration and STING down‐regulation with endocrine therapy resistance. In melanoma, Aung et al.^191^ developed compartment‐specific gene signatures (e.g., S100B) via GeoMx DSP, outperforming bulk RNA‐seq in predicting immunotherapy outcomes. Peyraud et al.,^171^ in NSCLC, identified mTLSs as immunotherapy response biomarkers, independent of PD‐L1 status. These spatially resolved biomarkers enhance patient stratification and guide precision therapeutic strategies.

In the context of immunotherapy resistance, spatial omics elucidates immune evasion mechanisms by mapping immune cell distribution and functional states within the TME. Monkman et al.,^167^ using CODEX in NSCLC, found Tregs enriched in stromal and tumour‐margin regions of non‐responders, spatially associated with monocytes and CD8⁺ T cells, suggesting Treg‐mediated immunosuppression as a resistance driver. Quek et al.,^166^ in melanoma, identified an ‘immune‐striving’ TME with peri‐tumoural lymphoid aggregates and low intra‐tumoural T‐cell infiltration, linked to immunotherapy resistance via CODEX. Yan et al.,^172^ in NSCLC, demonstrated that activated TLSs distal to tumours correlated with improved outcomes, while hypoxic TLSs near residual tumour cells were associated with poor immunotherapy responses, using GeoMx DSP and Visium. Naei et al.,^180^ in HNSCC, mapped macrophage–tumour cell barriers and immune aggregates with PhenoCycler, distinguishing responders from non‐responders to anti‐PD‐1 therapy. These insights reveal how spatial omics deciphers immune evasion by detailing immune landscapes and their functional contributions to therapy resistance.

The field is advancing at a remarkable pace, driven by *progress* in both experimental and computational frontiers. Research is rapidly moving beyond the spatial transcriptome to incorporate multiple layers of biological regulation, providing a more holistic understanding of CTR. Spatial epigenomics technologies now enable the mapping of chromatin accessibility and histone modifications at near‐single‐cell resolution, revealing how the epigenetic landscape shapes gene expression in complex tissues. Concurrently, spatial metabolomics holds immense promise for visualising the metabolic reprogramming and heterogeneity that allow cancer cells to adapt to therapeutic stress. The integration of these modalities is being realised through technologies like DBiT‐seq,^193^ and SPARC‐seq,^194^ Slide‐DNA‐seq,^195^ Slide‐TCR‐seq,^196^ SM‐Omics,^197^ Spatial CITE‐seq,^198^ Spatial ATAC–RNA‐seq,^199^ Spatial CUT&Tag–RNA‐seq^199^ and computational frameworks designed to align and interpret multi‐omics datasets.^200^, ^201^ Furthermore, the field is transitioning from two‐dimensional (2D) analysis to three‐dimensional (3D) spatial reconstruction, which overcomes the sampling biases of single tissue sections to provide a more complete picture of tumour architecture, immune infiltration and vascular networks. Tools like Spateo are enabling the creation of 3D spatiotemporal models that bridge macroscopic tissue changes with underlying molecular dynamics.^58^

Despite this rapid progress, several fundamental *limitations* remain, defining the next set of challenges for the field. While 3D reconstruction from serial sections is a major advance, these methods provide a static snapshot and do not yet capture the temporal dynamics of resistance as it evolves in a living system. The computational integration of disparate multi‐omics datasets, especially from unmatched samples, continues to be a formidable challenge requiring sophisticated algorithms. The ultimate ambition is to build a ‘digital twin’ of a tumour, which is a dynamic, 3D, multi‐omics model that can predict tumour evolution and response to therapy. Although this goal remains on the horizon, the ongoing convergence of spatial technologies and advanced computational modelling is charting a clear path forward. These innovations will not only continue to deepen our fundamental understanding of the mechanisms of CTR but are also poised to redefine precision medicine by enabling patient stratification and the design of novel therapeutic strategies based on the spatial biology of a tumour.

## AUTHOR CONTRIBUTIONS

**Yinghao Zhang**: investigation; writing—original draft and editing; writing—review; figure design. **Cheng Yang**: investigation; writing—review. **Xi Chen**: investigation; writing—original draft. **Liang Wu**: investigation; writing—original draft; writing—review. **Zhiyuan Yuan**: investigation; writing—original draft; writing—review; figure design. **Fan Zhang**: investigation; writing—review. **Bin‐Zhi Qian**: conceptualisation; funding acquisition; project administration; supervision; investigation; writing—original draft and editing; writing—review; figure design. All authors have read and approved the final version of the manuscript and agree with the order of authorship.

## CONFLICT OF INTEREST STATEMENT

The author declares that there is no conflict of interest that could be perceived as prejudicing the impartiality of the research reported.

## ETHICS STATEMENT

These issues are not applicable for this review.

## Data Availability

These issues are not applicable for this review.
